# A190 *HELICOBACTER PYLORI* INDUCES AN AUTO-REACTIVE PHENOTYPE IN PINK1 DEFICIENT MICE THAT CORRELATES WITH MOTOR DYSFUNCTION

**DOI:** 10.1093/jcag/gwad061.190

**Published:** 2024-02-14

**Authors:** A Kazanova, H Bessaiah, J Sung, C Gavino, J Pei, S Recinto, W Miller, L Burns, L Zhu, J Stratton, S Gruenheid

**Affiliations:** MIM, McGill University, Montreal, QC, Canada; MIM, McGill University, Montreal, QC, Canada; MIM, McGill University, Montreal, QC, Canada; MIM, McGill University, Montreal, QC, Canada; MIM, McGill University, Montreal, QC, Canada; MIM, McGill University, Montreal, QC, Canada; MIM, McGill University, Montreal, QC, Canada; McGill University, Montreal, QC, Canada; MIM, McGill University, Montreal, QC, Canada; MIM, McGill University, Montreal, QC, Canada; MIM, McGill University, Montreal, QC, Canada

## Abstract

**Background:**

Parkinson’s disease (PD) is a chronic neurodegenerative disorder. Several genetic predispositions, including the PTEN-induced kinase 1 (PINK1) mutation, have been implicated in early onset family cases. Besides the loss of dopaminergic neurons in the brain, PD patients have a unique immune phenotype that includes increased inflammation, blood serum, brain levels of proinflammatory cytokines, brain infiltration with cytotoxic CD8 T cells, and loss of regulatory T cells (Treg) and their anti-inflammatory phenotype.

The role of the gut microbiota and gastrointestinal infections are increasingly recognized as a cofactor in PD. One of pathogens associated with the risk of PD is *Helicobacter pylori*. The prevalence of this Gram-negative bacteria in PD patients is higher than in the general population and its eradication in PD patients improves motor function.

Loss of the PD-associated PINK1 alters induced Treg function *in vitro*. We previously have shown that in PINK1 knock-out (KO) mice, gut infection with Gram-negative bacteria, *Citrobacter rodentium*, induces mitochondrial antigen presentation (MitAP) to the CD8 T cells that later infiltrate the brain. In this model, PINK1 KO mice develop a Parkinson-like L-DOPA-responsive motor phenotype after four *C. rodentium* infections.

**Aims:**

Here we aimed to scrutinize potential role of the *H. pylori* infection in the induction of motor dysfunction and disbalanced immune tolerance in PINK1 KO mice.

**Methods:**

In addition to standard behavioural testing, at 2- and 6-months post-infection we performed a multiplex cytokine analysis of gastric homogenates and spectral flow cytometry of gastric lamina propria, mesenteric lymph nodes, blood, spleen, and brain infiltrating immunocytes. As well as *in vitro* immunocytes response and function assays to *H.pylori* exposure in PINK1 KO and wild-type (WT) littermate controls.

**Results:**

We show that infection with *H. pylori* causes a long-lasting inflammation in the stomach; and local and systemic immune phenotype that remains 6 months post-infection. This phenotype, though present in some WT infected mice, is higher in PINK1 KOs and similarly to PD patients includes a decrease in Treg proportion, FoxP3 downregulation in regulatory T cells, Gata3+ CD4 T cell loss, as well as increase of circulating mitochondrial antigen-specific CD8 T cells. Moreover, the immune phenotype in the *H. pylori* infected PINK1 KO mice correlates with motor dysfunction and CD8 T cell brain infiltration with no such association seen in the WT mice.

**Conclusions:**

These results provide insight to the gut-immunity-brain axis in the pathogenesis of Parkinson’s disease, and further investigate the role of Gram-negative bacteria in the establishment of immune tolerance-autoreactivity balance.

**Funding Agencies:**

ASAP

